# Association between eating behavior scores and obesity in Chilean children

**DOI:** 10.1186/1475-2891-10-108

**Published:** 2011-10-11

**Authors:** José L Santos, Judith A Ho-Urriola, Andrea González, Susan V Smalley, Patricia Domínguez-Vásquez, Rodrigo Cataldo, Ana M Obregón, Paola Amador, Gerardo Weisstaub, M Isabel Hodgson

**Affiliations:** 1 Department of Nutrition, Diabetes and Metabolism. School of Medicine. Pontificia Universidad Católica de Chile. Alameda 340. Santiago, Chile; 2 Instituto de Nutrición y Tecnología de los Alimentos (INTA). Universidad de Chile. Santiago, Chile

**Keywords:** feeding behavior, obesity, children

## Abstract

**Background:**

Inadequate eating behavior and physical inactivity contribute to the current epidemic of childhood obesity. The aim of this study was to assess the association between eating behavior scores and childhood obesity in Chilean children.

**Design and methods:**

We recruited 126 obese, 44 overweight and 124 normal-weight Chilean children (6-12 years-old; both genders) according to the International Obesity Task Force (IOTF) criteria. Eating behavior scores were calculated using the Child Eating Behavior Questionnaire (CEBQ). Factorial analysis in the culturally-adapted questionnaire for Chilean population was used to confirm the original eight-factor structure of CEBQ. The Cronbach's alpha statistic (>0.7 in most subscales) was used to assess internal consistency. Non-parametric methods were used to assess case-control associations.

**Results:**

Eating behavior scores were strongly associated with childhood obesity in Chilean children. Childhood obesity was directly associated with high scores in the subscales "enjoyment of food" (P < 0.0001), "emotional overeating" (P < 0.001) and "food responsiveness" (P < 0.0001). Food-avoidant subscales "satiety responsiveness" and "slowness in eating" were inversely associated with childhood obesity (P < 0.001). There was a graded relation between the magnitude of these eating behavior scores across groups of normal-weight, overweight and obesity groups.

**Conclusion:**

Our study shows a strong and graded association between specific eating behavior scores and childhood obesity in Chile.

## Background

Eating habits and inclinations to food are acquired in early childhood, representing behavior traits that may change over time according to personal experiences [1-3]. In addition to the broad social influences that clearly have an impact on dietary intake, it is accepted that family influences (both common environment and genetic inheritance), play a role in determining food intake patterns, eating behavior and childhood obesity [4,5].

A number of psychometric instruments have been used to assess eating behavior traits in children and adults for predicting risk of eating disorders and body weight-related problems. Some questionnaires are useful to complete food intake information and to assess feeding practices such as for example, eating in front of the TV or purchasing habits [6]. Eating behavior scores obtained from questionnaires in children represent subjective information that may change over time. However, they have advantages over dietary intake reports since they can be answered by one informant (usually, the mother), who has a near-complete observational access to her child in a wide range of situations [7,8]. Additionally, it has been shown that eating behavior scores using psychometric tools show a strong and graded association with childhood obesity [9]. The CEBQ is generally regarded as one of the most comprehensive instruments in assessing children's eating behavior. It was developed and validated in the United Kingdom [10], and recently has been validated in other European studies [11].

Chile, as well as other countries in Latin-America, has suffered a nutrition transition in the last 2 decades, with changes in life-styles towards an increased consumption of high-energy foods and sedentary habits. What makes Chile an interesting example in nutrition transition is that this process occurred at a much faster rate than other countries in the region, with a rapid decline in under-nutrition (from 16% in 1985 to <1% in 1995) and stunting (low-height-for-age), from 10% to 2% in the same period [12,13]. At the same time, there has been an increase in the prevalence of obesity (more than three-fold change in the last 15 years). As a consequence of this situation, it is possible that attitudes and practices of many Chilean mothers on feeding and nutrition are somehow still dominated by a sense of protection against childhood undernutrition instead of preventing childhood obesity [14-16]. In this context, it is important to identify behavioral factors that affect weight excess during childhood beyond focusing on the amount and type of foods usually consumed. Additionally, a better understanding of the link between eating behavior and childhood obesity is also of interest with the purpose of designing interventions based on behavioral modifications. Therefore, the aim of the present study was to assess the association between childhood eating behavior scores and obesity in obese, overweight and normal-weight Chilean children.

## Design, Subjects and Methods

### Design and subjects

We conducted a case-control study in the city of Santiago (Chile) with n = 126 obese and n = 124 normal-weight children (6-12 years-old; both genders; 50% boys), classified according to the IOTF criteria [17] during the years 2006 to 2009. Cases and controls were selected from the outpatient unit of the Pediatrics Department at the INTA (Institute of Nutrition and Food Technology, University of Chile), from outpatient unit of the School of Medicine of the Pontificia Universidad Católica de Chile and from public schools through an open invitation to families. All participants received nutritional advice by a nutritional expert and educational brochures and documents promoting healthy eating behaviors and lifestyles. The study was approved by the Ethics Committee of the University of Chile and the Pontificia Universidad Católica de Chile. Written informed consents were obtained from parents or guardians of the children. An additional group of n = 44 overweight children according IOTF criteria (mean age of 9.6 years; 45.4% boys) was recruited to assess a possible graded association between eating behavior scores and obesity status.

### Anthropometric measurements

Anthropometric measurements were carried out using standardized techniques by trained personnel [18,19]. Height and weight were measured in light clothing, and Body Mass Index (BMI) was calculated as weight in kilograms divided by the square of height in meters (kg/m^2^). Waist circumference was measured using a non-elastic tape just above the uppermost lateral border of iliac crest, at the end of a normal expiration. The pubertal stage (development of breast, genital, and pubic hair) was documented in all children according to the classification of Tanner through self-assessment using pictures [20-23]. Nutritional status classification was based on BMI for age and sex, using the cut-off points of the International Obesity Task Force [17].

### Child Eating Behavior Questionnaire (CEBQ)

CEBQ is a 35-item questionnaire that evaluates eight subscales of eating behavior: Food Responsiveness (FR; 5 items), Enjoyment of Food (EF; 4 items), Emotional Over-Eating (EOE; 4 items), Desire to Drink (DD; 3 items), Slowness in Eating (SE; 4 items), Satiety Responsiveness (SR; 5 items), Food Fussiness (FF; 6 items) and Emotional Under-Eating (EUE; 4 items) [10]. The first four subscales (EF, FR, EOE and DD) are "food-approach" subscales that indicate positive inclinations for eating while the other four subscales (SR, SE, FF and EUE) are considered as "food-avoidant" subscales related to negative inclinations to food intake. EF and FR reflect different aspects of excessive responsiveness to external food cues. EOE and EUE measure an increase or a decrease in eating in response to a range of negative emotions, such as anger, loneliness, or anxiety. DD reflects the inclination of children to drink frequently, sometimes associated with an increased intake of sugar-sweetened drinks. SR represents the ability of a child to reduce food intake after eating to regulate its energy intake [24]. High scores of SE mean a reduction in eating rate as a consequence of lack of enjoyment and interest in food. Finally, FF is related with a rejection of a substantial amount of novel and common foods, narrowing the range of the variety of consumed foods. Each item was answered in a Likert-type scale with possible scores from 1 to 5, where 1 is complete absence and 5 the highest intensity of the specific eating behavior.

CEBQ was translated to Chilean-Spanish language through a direct and reverse translation procedure, and subsequently adapted for the Chilean culture through the assessment of responses from ten child-mother duos [25]. Direct interview with the mothers in home visits or outpatient's facilities were carried out in order to calculate eating behavior scores in children.

### Internal consistency of CEBQ subscales

It has been reported that CEBQ shows adequate internal consistency, test-retest reliability, and stability over time [26]. In our study, internal consistency for each subscale was assessed through the α-Cronbach statistic: "food responsiveness" (0.89), "enjoyment of food" (0.79), "emotional over-eating" (0.81) and "desire to drink" (0.70), "slowness in eating" (0.82), "satiety responsiveness" (0.73), "emotional under-eating" (0.57), "food fussiness" (0.82) and the combined "satiety-slowness index" (0.84).

### CEBQ factor structure

In the factorial analysis, seven factors with eigenvalues above 1.0 explaining 62% of total variance were identified using the principal component extraction method (command "factor", option "pcf" in STATA 11.0 package). The scree plot [See Additional File 1] shows that either the eight-factor or the seven-factor solutions are both acceptable given the slope of the chart. After varimax rotation, loadings of different items are shown in Table 1. It is important to mention that some identified factors in our study have shown a substantial degree of overlapping, especially SR with SE. As previously described, a subscale can be created with the combination of satiety responsiveness and slowness in eating (the combined "satiety-slowness index"). However, we have preferred to maintain the original factors in the case-control association analysis to allow comparison with other studies [[10,11] and [27]]. The factor analysis was carried out in all the participants of the study (n = 294; 126 obese, 124 normal-weight and 44 overweight children).

**Table 1:** Factor loadings for CEBQ items estimated from the principal components analysis

Number Factor**		Factor Loading	Communality
**Factor 1** **26.4%**	**Food Responsiveness**		
	My child is always asking for food	0.76	0.69
	Given the choice, my child would eat most of the time	0.87	0.80
	Even if my child is full up she finds room to eat her favorite food	0.62	0.55
	If allowed to, my child would eat too much	0.83	0.75
	If given the chance, my child would always have food in her mouth	0.78	0.73
**Factor 4** **5.3%**	**Enjoyment of Food**		
	My child is interested in food	0.69	0.65
	My child looks forward to mealtimes^a^	0.44	0.61
	My child enjoys eating	0.75	0.68
	My child loves food	0.62	0.62
**Factor 5** **4.9%**	**Emotional Over Eating**		
	My child eats more when worried	0.71	0.65
	My child eats more when annoyed	0.70	0.70
	My child eats more when she has nothing else to do^b^	0.35	0.54
	My child eats more when anxious	0.69	0.73
**Factor 6** **3.7%**	**Desire for Drinks**		
	My child is always asking for a drink	0.83	0.73
	If given the chance, my child would always be having a drink	0.86	0.77
	If given the chance, my child would drink continuously throughout the day	0.62	0.80
**Factor 7** **3.1%**	**Emotional Under Eating**		
	My child eats less when angry	0.70	0.57
	My child eats less when she is tired	0.18	0.85
	My child eats less when upset	0.80	0.70
	My child eats more when she is happy	0.59	0.54
**Factor 3** **7.3%**	**Food Fussiness**		
	My child refuses new foods at first	0.84	0.73
	My child enjoys a wide variety of foods (Reverse item)	0.31	0.56
	My child is interested in tasting food she hasn't tasted before (Reverse item)	0.80	0.68
	My child decides that she doesn't like a food even without tasting it	0.79	0.69
	My child is difficult to please with meals	0.59	0.50
	My child enjoys tasting new foods (Reverse item)	0.81	0.68
**Factor 2** **11.1%**	**Slowness in Eating/Satiety Responsiveness**		
	My child finishes her meal quickly (Reverse item)	0.70	0.65
	My child takes more than 30 minutes to finish a meal	0.78	0.65
	My child eats less when angry	0.76	0.71
	My child eats more and more slowly during the course of a meal	0.75	0.60
	My child leaves food on her plate at the end of a meal	0.54	0.56
	My child gets full up easily	0.30	0.54
	My child gets full before her meal is finished	0.56	0.50
	My child cannot eat a meal if she has had a snack just before	0.33	0.42
	My child has a big appetite (Reverse item)	0.21	0.62

** Number Factor given by Stata Program on the principal component factor analysis.

^a b ^Factor loading was 0.61 in factor 1

### Graphical representation of CEBQ scores

We have used radar charts to graphically represent the multidimensionality of childhood eating behavior measured through CEBQ scores [See Additional File 2]. In this graphic, scores for each subscale were projected in such a way that the upper part of the chart shows the four "food-approach" subscales related with positive inclinations to food intake while the lower part shows the four "food-avoidant" subscales. The sum of "food-approach" and "food-avoidant" subscales can be used as a rough combined index of "food approach" and "food avoidant" behaviors respectively.

### Statistical analysis for the case-control study

Summary statistics of CEBQ scores are shown as quartiles. Non-parametric statistical tests (Mann-Whitney test, Kruskal-Wallis test or Spearman correlation coefficients) were used to assess association between study variables. Additional association analyses between obesity and CEQB scores were carried out by creating 1:1 age-gender matched datasets for case-control comparisons. All statistical analyses were carried out with the STATA 11.0 package http://www.stata.com.

## Results

**Figure 1:**
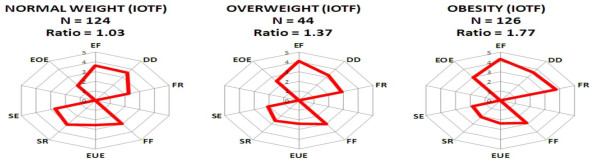
**Childhood eating behavior scores (CEBQ) and obesity status in Chilean children**. Median of the eating behavior scores in normal weight, overweight and obese children according to IOTF criteria. The "ratio" refers to the quotient between the sums of scores of the "food-approach" subscales divided by the sum of the scores of the "food-avoidant" subscales.

**Figure 2:**
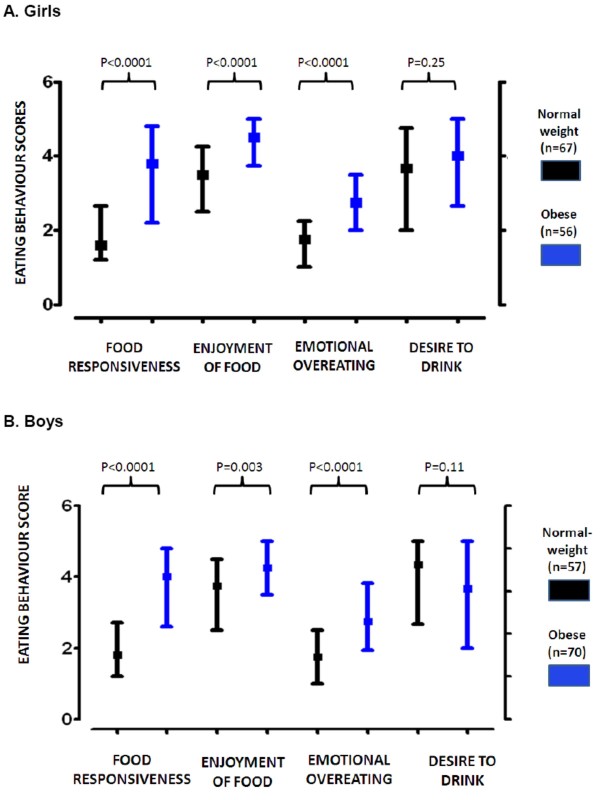
**"Food approach" CEBQ subscales of Chilean children (6-12 yrs)**. Results of case-control analyses (obese versus normal-weight) using the Mann-Whitney test. A: the results are presented as quartiles of eating behavior scores in girls (age 6-12 y). B: quartiles of eating behavior in boys (age 6-12 y). Statistical analyses indicated that positive inclinations were strongly associated with childhood obesity in all the "food approach" subscales except with "desire to drink" subscale.

**Figure 3:**
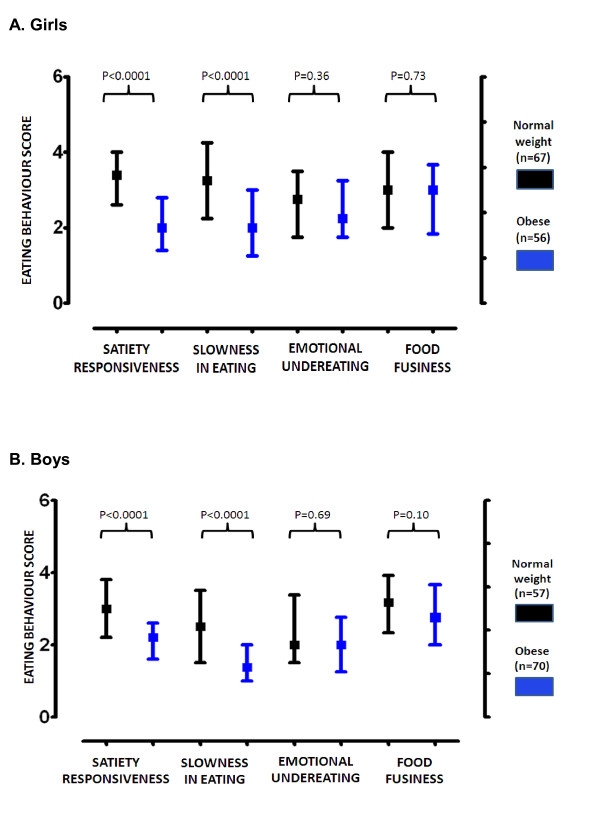
**"Food-avoidant" CEBQ subscales of Chilean children (6-12 yrs)**. A: the results are presented as quartiles of eating behavior scores in girls (age 6-12 y). B: quartiles of eating behavior in boys (age 6-12 y). In the "food avoidant" subscales, "satiety responsiveness" and "slowness in eating" were significantly and inversely associated with childhood obesity (Mann-Whitney test).

Table 2 shows the anthropometric characteristics of our study group. Figure 1 shows multivariate radar charts showing the median values of eating behavior scores in normal-weight, overweight and obese children according to IOTF categories. Eating behavior scores were strongly associated with childhood obesity in Chilean children [Figures 2 and 3]. Eating behavior scores indicating positive inclinations to higher intake were strongly associated with childhood obesity in the subscales "food responsiveness" (P < 0.0001 in both genders), "enjoyment of food" (P < 0.0001 in girls and P = 0.003 in boys) and "emotional overeating" (P < 0.0001 in both genders). No significant associations with obesity was found for "desire to drink" (P = 0.25 in girls and P = 0.11 in boys). On the other hand, food-avoidant subscales "satiety responsiveness" and "slowness in eating" were significantly and inversely associated with childhood obesity (P < 0.0001 in both genders). "Food-fussiness" (P = 0.73 in girls and P = 0.1 in boys) and "emotional under-eating" (P = 0.36 in girls and P = 0.69 in boys) were similar in obese versus normal-weight children. Significant association between obesity status and the combined "satiety-slowness index" was also found (P < 0.0001).

**Table 2:** Anthropometric variables in normal-weight, overweight and obese Chilean children (IOTF criteria)

	Girls			Boys		
	Normal-weight (n = 67)	Overweight (n = 24)	Obesity (n = 56)	Normal-weight (n = 57)	Overweight (n = 20)	Obesity (n = 70)
**Age (years)**	10.6 ± 1.4	9.4 ± 1.4	8.9 ± 1.7	10.6 ± 1.4	9.9 ± 1.4	9.3 ± 1.7
**BMI Z-score**	-0.1 ± 0.6	1.9 ± 0.9	3.7 ± 0.9	-0.1 ± 0.7	2.6 ± 0.9	4.6 ± 1.4
**Weight Z-score**	-0.4 ± 0.8	1.3 ± 0.5	2.2 ± 0.4	-0.4 ± 0.9	1.6 ± 0.5	2.4 ± 0.4
**Height Z-score**	-0.01 ± 0.3	0.2 ± 0.9	0.6 ± 0.7	-0.3 ± 0.9	0.4 ± 0.9	0.9 ± 0.9
**Waist-to-height ratio**	0.4 ± 0.03	0.5 ± 0.03	0.6 ± 0.04	0.5 ± 0.03	0.6 ± 0.03	0.6 ± 0.04

Results are expressed as the means ± SD.

We have not found overall significant differences in "food-approach" CEBQ scores by gender (P = 0.58 for FR; P = 0.96 for EF, P = 0.44 for EOE and P = 0.43 for DD). However, we found significantly higher scores for "food-avoidant" CEBQ subscales in girls compared to boys, as in SE (P < 0.001), SR (P = 0.04) and EUE (P = 0.03), without finding significant differences for FF (P = 0.99). As a consequence of this, the sum of the "food-approach" CEBQ scores were not significantly different when comparing boys and girls (P = 0.58) [See Additional File 3], while strong significant differences were found when comparing boys and girls in relation to the "food-avoidant" CEBQ scores (P = 0.006) [See Additional File 4]. When using the summation of "food-approach" and "food-avoidant" subscales, we observed a graded and strong association with obesity status (normal-weight, overweight and obesity) (P < 0.0001). Matched analysis for case-control yielded essentially the same results than the unmatched case-control analysis (data not shown).

There was no significant association between age and "food-approach" CEBQ scores when girls and boys were evaluated separately, except for a inverse relation between age and scores of EOE in girls (rho = -0.20; P = 0.01). We also found a direct association between age and EUE scores in girls (rho = 0.24; P = 0.003). When taking the sum of "food-approach" or "food-avoidant" CEBQ scores by gender, a significant direct association was only found between age and the sum of "food-avoidant" CEBQ scores in girls (rho = 0.18; P = 0.03).

## Discussion

It has been described that eating behavior, measured through CEBQ in children, is a relatively stable trait over time, showing a good reproducibility and high internal consistency [28]. A clear and graded association between CEBQ scores and BMI has been reported previously [10,11,27]. The present study confirms the existence of such association in Chilean children, especially in relation to the positive associations between obesity and "food-approach" ("pro-intake") subscales such as EF, FR and EOE. These results are similar to previous studies showing that children with increased BMI are highly responsive to environmental food cues. The inverse associations between body weight and scores of "food-avoidant" ("anti-intake") subscales such as SE and SR are similar to other studies. The CEBQ subscales DD, EUE and FF showed no association with childhood obesity [11,27].

The original 8-factor structure was not perfectly replicated in our study since an important degree of overlapping has been found between SE and SR [29]. However, we have analyzed the eight original factors for case-control associations to allow for comparisons with other studies [10,11,27]. The importance of focusing on behavioral traits is that eating behavior is susceptible to modification through adequate interventions to prevent and/or treat childhood obesity. In this context, changes in CEBQ scores can be also used to assess the effectiveness of such preventive/therapeutic actions [30]. The results of our study indicate that the population of children under the age of 12 could be the target of intervention programs (education provided to families, promoting healthy diets and lifestyles) to improve the nutritional condition and the control of non-communicable diseases. On the other hand, the genetic influence on human feeding behavior has also been evaluated through studies of twins. TEDS (Twin Early Development Study) evaluated eating behavior at the age of 11 in monozygotic (MZ) and dizygotic (DZ) twins born in the United Kingdom through the questionnaire CEBQ [31]. In addition of finding a high heritability for the BMI in childhood, a significantly higher correlation in MZ twins in relation to DZ twins in the subscales of enjoyment of food and satiety response was found in TEDS [31].

We have been evaluating the best way to represent graphically the multidimensionality of childhood eating behavior. After evaluating different options of multivariate charts, we have arrived to the so-called multivariate radar charts. We have used this type of chart to report child eating behavior to Chilean families, who understand it very well and consider useful. This type of chart defines two polygons: the upper polygon representing positive attitudes to food intake, and the lower polygon for negative attitudes to food intake. Although the ratio of areas of the upper and lower polygons might be a considered as a gross index for general children eating behavior, this index is in fact not adequate because the shapes and areas of the polygons are modified by only changing the order of the subscales. On the contrary, the sum of either "food-approach" or "food-avoidant" scores are not affected by this distorting effect and their ratio is used in Figure 1 as a summary index. In spite of the limitations commented above, we believe that radar charts are useful for health professionals in visualizing and interpreting children eating behavior.

There are few publications that have evaluated eating behavior in children through the CEBQ [10,11,27]. To our knowledge, this study is the first to assess the factor structure of the CEBQ and association with BMI in a Latin America population. Some advantages of our study were that children's weight and height were measured directly and not parentally reported and all interviews were conducted face-to-face by trained personnel. There are a number of limitations in our research derived from its limited sample size and the cross-sectional nature of this study. Significant associations detected in this study may in fact be due to reverse causation in which the disease (childhood obesity) caused mothers to change their perception of child's eating behavior. On the other hand, there is an inherent uncertainty related to the measurement of subjective eating behavior in humans through questionnaires, in contrast to direct measurements of eating behavior [32].

## Conclusion

Our data of Chilean children confirmed the strong association between specific eating behavior scores and childhood obesity found in previous studies. We found a direct association between obesity and "food-approach" subscales such as "enjoyment of food", "food responsiveness" and "emotional over-eating". On the other hand, scores of "food-avoidant" subscales "satiety responsiveness" and "slowness in eating") were inversely associated with body weight.

## Abbreviations

BMI: body mass index; CEBQ: Child Eating Behavior Questionnaire; DZ: dizygotic; EI: energy intake; EF: enjoyment of food; EOE: emotional over-eating; EUE: emotional under-eating; FF: food fussiness; FR: food responsiveness; IOTF: International Obesity Task Force; MZ: monozygotic; SE: slowness in eating; SR: satiety responsiveness; TEE: total energy expenditure; TEDS: Twin Early Development Study.

## Competing interests

The authors declare that they have no competing interests.

## Authors' contributions

JLS designed, supervised and participated in the conduction of the research, JLS, JHU, MIH, AG, SS, P.A., PDV, GW, RC and AMO conducted the research, JLS, JHU and MIH analyzed the data and wrote the paper. JLS and M.I.H. had primary responsibility for the final content. All authors have read and approved the final manuscript.

## Supplementary Material

Additional file 1 **Scree-plot for the identification of CEBQ factors in Chilean children 6 - 12 years-old**. Eight factors were identified with eigenvalue > 1.0 using factor analysis with the principal component extraction method. The scree plot shows that either the eight-factor or the seven-factor solutions are both acceptable given the slope of the chart. Click here for file

Additional file 2 **Multivariate radar chart representing the information of the Child Eating Behavior Questionnaire (CEBQ)**. In this graphic, scores for each subscale were projected in such a way that the upper part of the chart shows the four "food-approach" subscales related with positive inclinations to food intake while the lower part shows the four "food-avoidant" subscales. Click here for file

Additional file 3 **Weighed sum of "food approach" CEBQ subscales in Chilean children from 6 - 12 years**. The "Food-approach" subscales: FR (Food Responsiveness), EF (Enjoyment of Food), EOE (Emotional Over-Eating) and DD (Desire to Drink), scores were not significantly different when comparing boys and girls (P = 0.58). Click here for file

Additional file 4 **Weighed sum of "food avoidant" CEBQ subscales in Chilean children from 6 -12 years**. The "Food-avoidant" subscales: SE (Slowness in Eating), SR (Satiety Responsiveness), FF (Food Fussiness) and EUE (Emotional Under-Eating), a strong significant differences were found when comparing boys and girls in relation to the "food-avoidant" CEBQ scores (P = 0.006). Click here for file
