# Supplementary figures and images for: Association between eating behavior scores and obesity in Chilean children
Source: Nutr J. 2011 Oct 11;10:108. doi: 10.1186/1475-2891-10-108 (PMC3213088; doi:10.1186/1475-2891-10-108)

Additional File 1

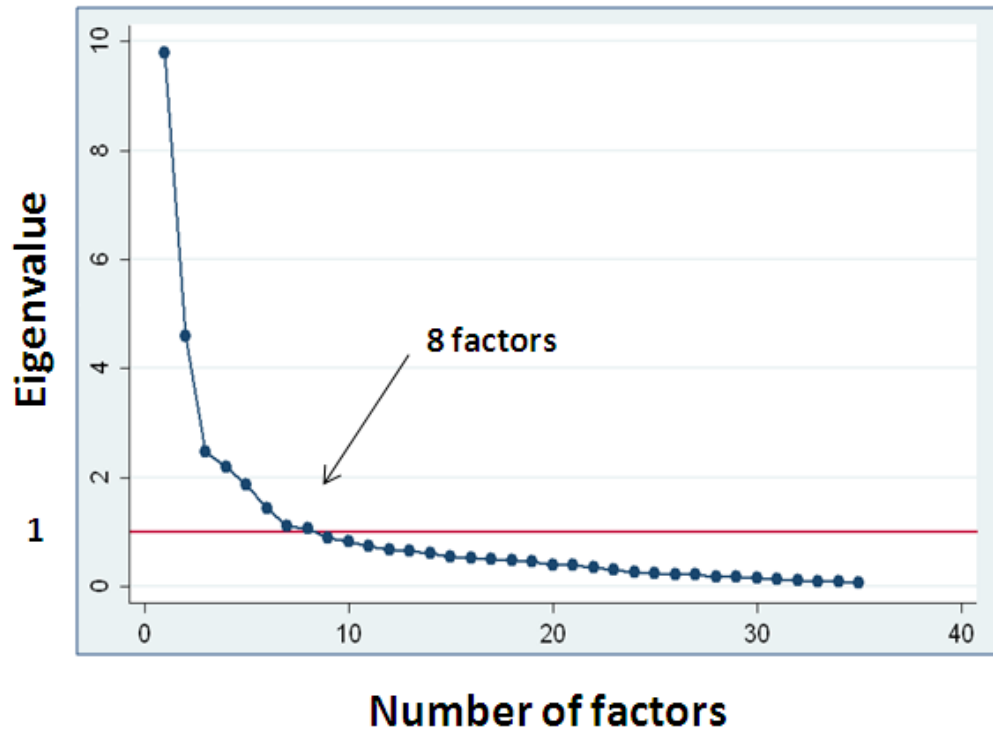

Supplement: Additional file 1 — Scree-plot for the identification of CEBQ factors in Chilean children 6 - 12 years-old. Eight factors were identified with eigenvalue > 1.0 using factor analysis with the principal component extraction method. The scree plot shows that either the eight-factor or the seven-factor solutions are both acceptable given the slope of the chart. [file 1475-2891-10-108-S1.PDF]

Additional File 2

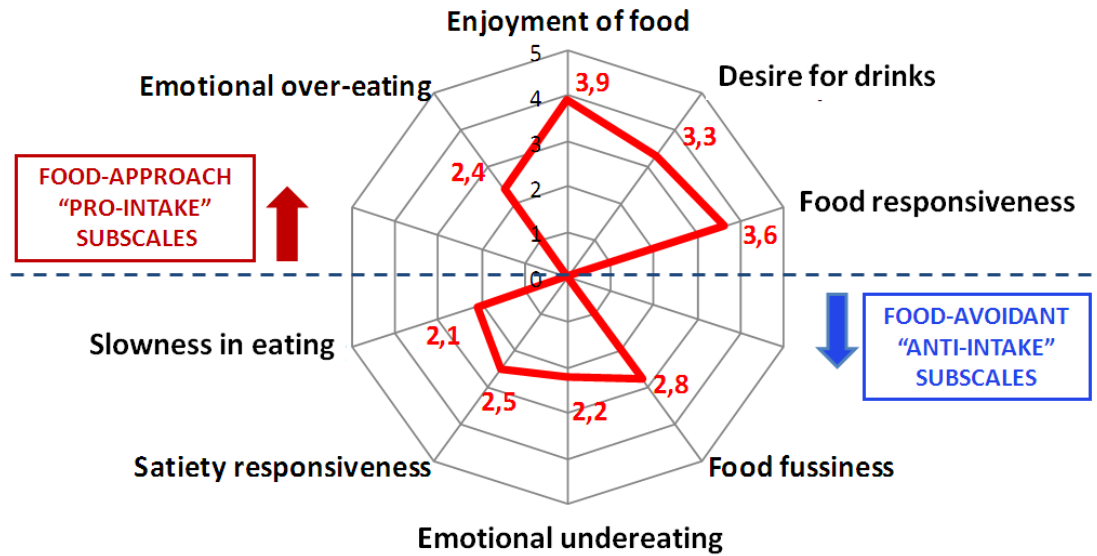

Supplement: Additional file 2 — Multivariate radar chart representing the information of the Child Eating Behavior Questionnaire (CEBQ). In this graphic, scores for each subscale were projected in such a way that the upper part of the chart shows the four "food-approach" subscales related with positive inclinations to food intake while the lower part shows the four "food-avoidant" subscales. [file 1475-2891-10-108-S2.PDF]

Additional File 3

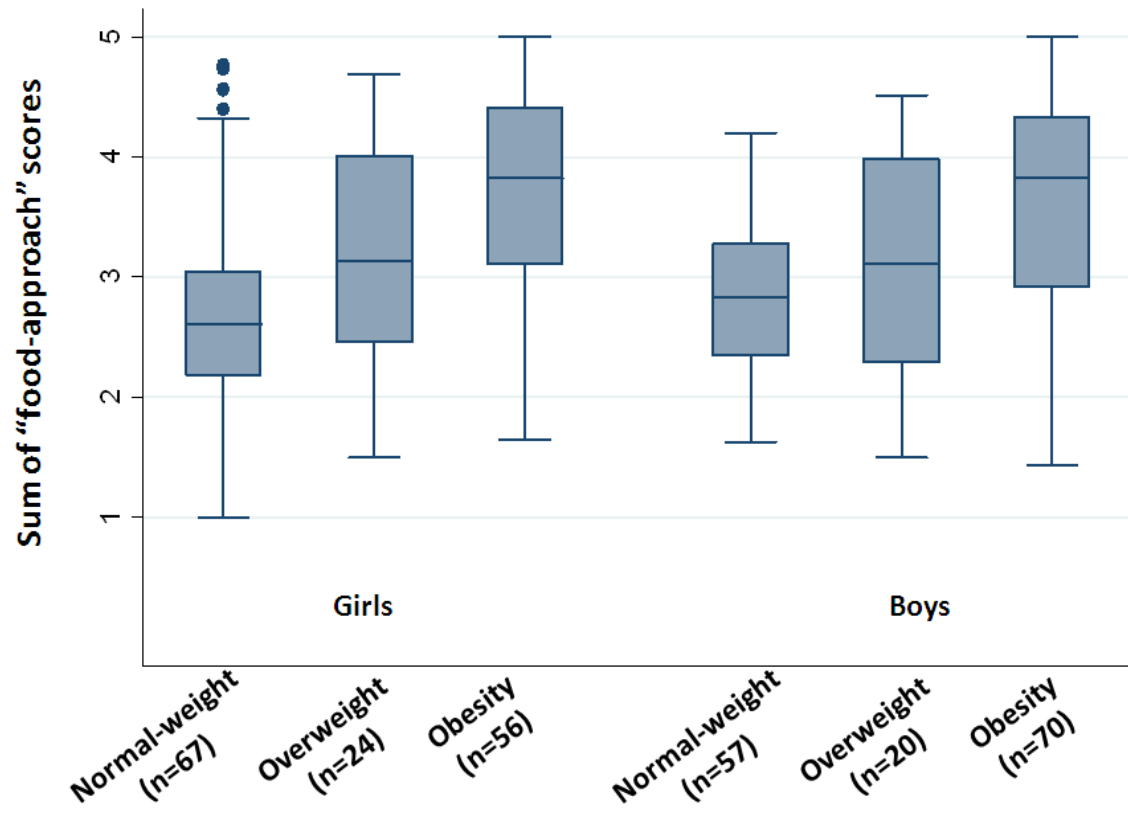

Supplement: Additional file 3 — Weighed sum of "food approach" CEBQ subscales in Chilean children from 6 - 12 years. The "Food-approach" subscales: FR (Food Responsiveness), EF (Enjoyment of Food), EOE (Emotional Over-Eating) and DD (Desire to Drink), scores were not significantly different when comparing boys and girls (P = 0.58). [file 1475-2891-10-108-S3.PDF]

Additional File 4

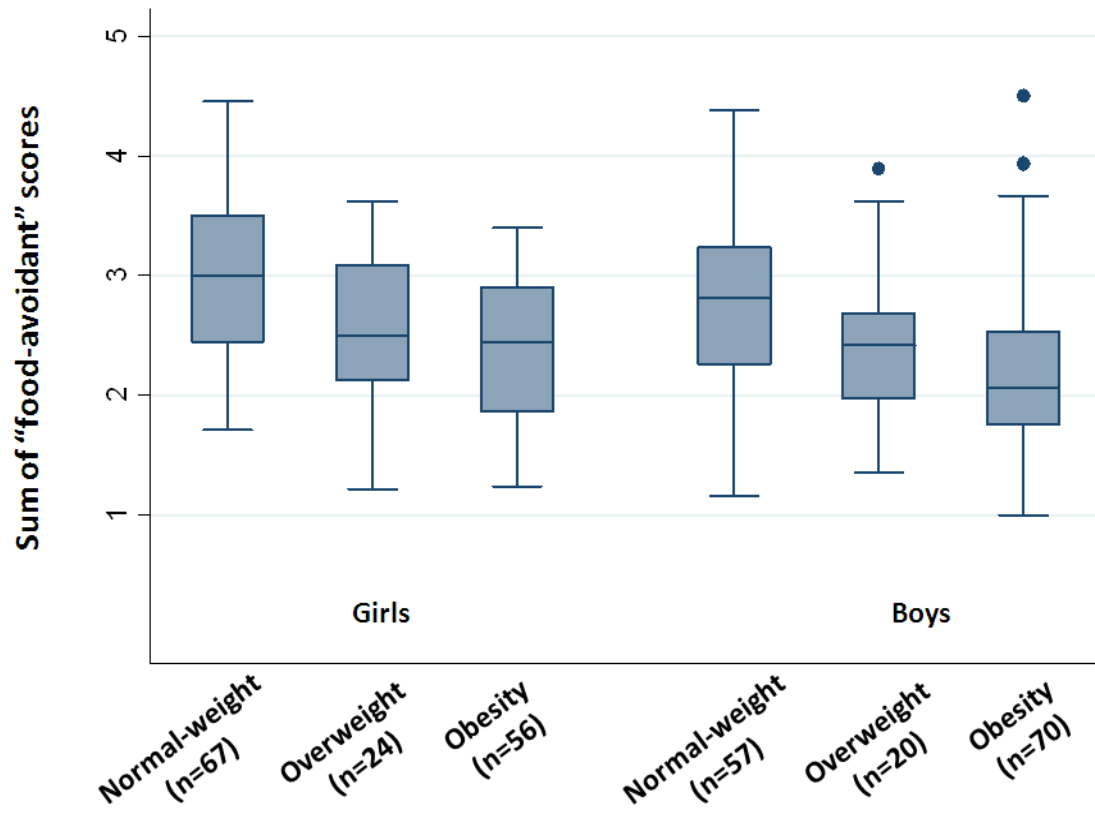

Supplement: Additional file 4 — Weighed sum of "food avoidant" CEBQ subscales in Chilean children from 6 -12 years. The "Food-avoidant" subscales: SE (Slowness in Eating), SR (Satiety Responsiveness), FF (Food Fussiness) and EUE (Emotional Under-Eating), a strong significant differences were found when comparing boys and girls in relation to the "food-avoidant" CEBQ scores (P = 0.006). [file 1475-2891-10-108-S4.PDF]
